# A systematic review of the immuno-inflammatory dysfunction secondary to viral hemorrhagic fevers; Ebola and Lassa fever

**DOI:** 10.1371/journal.pntd.0013230

**Published:** 2025-06-25

**Authors:** Samuel Ficenec, Nell Bond, Jerry Zifodya, John Schieffelin

**Affiliations:** 1 Department of Internal Medicine, Tulane University School of Medicine, New Orleans, Louisiana, United States of America; 2 Section of Infectious Diseases, Department of Pediatric Medicine, Tulane University School of Medicine, New Orleans, Louisiana, United States of America; 3 Section of Pulmonary and Critical Care Medicine, Department of Internal Medicine, Tulane University School of Medicine, New Orleans, Louisiana, United States of America; WRAIR, UNITED STATES OF AMERICA

## Abstract

The viral hemorrhagic fevers Ebola and Lassa fever are endemic to Sub-Saharan Africa. Both viruses are characterized by high case fatality risk and lifelong debilitating sequelae including blindness and deafness. However, despite these findings the mechanisms of disease and pathogenesis through which these viruses act remain poorly understood. The objective of this systematic review was to synthesize known data regarding both acute and chronic immune-inflammatory dysfunction. A comprehensive search strategy was conducted from July 2022- August 2024. A total of 1,587 articles were identified and evaluated for inclusion. In total 49 Ebola specific and 31 Lassa fever articles were included in this review. The results of this study found considerable dysregulation in immune-inflammatory homeostasis. Specifically, Ebola was found to induce increased concentrations of molecules associated with immune cell recruitment and migration during acute disease. In addition, the virus led to reduction in innate cell populations and expansion of T-cell population frequencies across disease outcomes. Studies of Lassa fever also demonstrated considerable immune dysregulation. However, given the relative lack of studies the exact mechanism of disease is unclear. Among disease survivors, both viruses demonstrate persistent chronic immune dysregulation years following disease onset. However, associating these findings with post-viral syndromes is controversial.

## Author summary

The Ebola and Lassa fever viruses are both causative agents of hemorrhagic fevers in Sub-Saharan Africa. Despite garnering global attention during the West African Ebola Epidemic of 2014 the mechanisms through which these viruses cause disease and their immunopathology remain poorly understood. This is problematic as both continue to plague the region through new epidemics, re-emergence of disease in those previously infected, and devastating Post-viral syndromes. Both viruses are rare diseases limiting the ability of researchers to generate robust data which hinders the understanding of both the acute and convalescent phases of disease. This systematic review synthesizes the known evidence of immune dysfunction and provides further analysis to generate more generalizable data to aid in identifying key pathways in the immune response. In this way, this work may lead to the development of therapeutics to reduce disease severity, improve survival, and the prevention and treatment of convalescent post-viral syndromes.

## Introduction

Viral infections can have profound impacts on quality of life. Most notably, the SARS-COV-2 (COVID-19) pandemic led to an unprecedented 774,889,074 infections [1]. Among the 767,850,451 survivors, approximately 10% or 77 million individuals developed post-viral sequelae [1,2]. The exact mechanisms through which these symptoms develop are poorly understood. However an underlying immune dysfunction or disruption in immuno-inflammatory homeostasis is thought to contribute to these post-viral sequelae termed Long Haul Covid [3,4]. Although the symptoms of Long Haul Covid have garnered much attention, post-viral syndromes are not isolated to this virus alone.

Ebola and Lassa fever (LF), both hemorrhagic fever viruses endemic to Sub-Saharan Africa, are known to cause severe post-viral syndromes. Post-Ebola Syndrome (PES) has been associated with symptoms including blindness, hearing loss, musculoskeletal pain, and abdominal pain, while survivors of Lassa fever (LF) have reported sequelae including deafness, ataxia, tremors, vision impairment, and renal disease [5–10]. In addition, the low resource environment in which these infections occur leaves survivors with a limited ability to seek care and prevent progression, and further morbidity, and mortality related to these diseases and their sequelae. In contrast to COVID-19, up to 90% and 30% of survivors of Ebola and LF go on to develop post-viral syndromes, respectively [8,10–12]. As the number of infections secondary to the Ebola and LF viruses increase, a more thorough understanding of pathogenesis is paramount to prevent further reductions in quality of life, morbidity, and mortality among hemorrhagic fever survivors.

There are limited data on potential mechanisms through which these viruses cause clinical disease. However, animal model and in-vitro studies of both viruses have demonstrated dysregulation of immune responses and a collapse of cell populations during the acute phase of illness [13–20]. Evidence has suggested that these findings may be chronic and persist through convalescence [21]. However, these studies are scarce and limited in their ability to show the full spectrum of immune dysfunction following infection.

The objective of this review is to synthesize the data regarding changes to the immuno-inflammatory system following either Ebola or Lassa virus infection among survivors and fatal cases of disease. Here, we describe what is known regarding immuno-inflammatory dysfunction and identify gaps in knowledge. We propose areas of research to increase understanding of pathophysiology and potentially lead to new treatment modalities for acute symptoms and sequelae Ebola and LF.

## Methods

A comprehensive electronic search strategy and systematic review was conducted from July 2022 to August 2024 to identify studies published and indexed in MEDLINE, EMBASE, and PubMed. All articles that had conducted studies of human subjects that were infected with the Ebola or Lassa fever viruses that included evaluations of lymphocytes, leukocytes, neutrophils, macrophages and monocytic cells, antibodies or measurement of immune cell mediators, cytokines, or biomarkers were considered for inclusion in this manuscript. All study designs and publication dates that generated primary data including case studies or case series were considered. This study was not limited to a geographic region and was conducted globally. Articles that were written in a language other than English were translated using electronic tools. The literature search strategy included key terms or medical subject headings (MeSH), synonyms, and Boolean operators. Key terms utilized for this search included the following: Ebola, Ebola Virus Disease, Post-Ebola Syndrome, Ebolavirus, Hemorrhagic Fever, Lassa fever virus, Lassa fever, Lassa virus, sequelae, post-viral syndrome, immune dysfunction, immune deficiency, T-lymphocyte, T-cell, B-cell, B-lymphocyte, immunology, immune response, immune phenotype, cytokine, biomarker, inflammatory, and inflammation. Abstracts and titles of all retrieved studies were imported into the Covidence Systematic Review Management system. Three study authors were responsible for screening titles and abstracts. If articles met study criteria and included data related to immunologic or inflammatory dysfunction the full text of each article was obtained. In areas of disagreement authors discussed and the final decision regarding inclusion was made by the senior author. Following decisions on inclusion the full text of all relevant studies was obtained. To ensure robustness of this systematic review the reference lists of included studies were reviewed to identify additional studies for consideration. Data abstraction was performed by a single author. This author met with the review team on a bi-monthly basis to review collected data and ensure quality control. Articles were excluded if they were conducted on non-human subjects, did not discuss or report laboratory findings related to immune-inflammatory dysfunction, or discussed disease caused by viruses other than the Ebola or Lassa hemorrhagic fever viruses. Articles which discussed confirmed cases of co-infection such as LF and malaria were also excluded to limit potential confounding. In this systematic literature review, a formal risk of bias assessment was not performed due to the rarity of diseases under investigation and the inherent limitations of the available literature. Given the limited number of studies, excluding certain articles based on a formal risk of bias evaluation may have further restricted available evidence, thereby reducing the strength and comprehensiveness of this manuscript.

Data regarding the cause of infection, demographics, biomarkers and cytokines, innate and adaptive immune cell population frequencies, responses to viral antigens, antibodies and antibody function, and disease outcomes were extracted, aggregated by the respective virus, and tabulated in Microsoft Excel software (Redmond, WA). Data were loaded into R statistical software version 3.6.2 evaluated within the RStudio environment. Data regarding biomarkers were grouped according to all functions and comparisons made between groups of biomarkers and fatal infections and survivors. Similarly, data regarding cell populations was grouped by cell type. Comparisons were made among data with the use of Kruskal-Wallis or Fisher’s Exact Tests to evaluate for differences between groups, pairwise comparisons between fatal cases of infection and survivors were completed using Wilcox Rank Sum test while controlling for multiple comparisons with Bonferonni Corrections where applicable. Each observed change in biomarker was assigned a numerical value (Decreased = -1, No Difference = 0, and Increased = 1). Multinomial ordinal logistic regression modelling was then completed to evaluate for the strength and the direction of molecular biomarker changes following EBOV or LASV infection by calculating odds ratio (OR) and 95% confidence intervals (95%CI). The biomarker functional group which was found to be the most neutral or least likely to have changed in concentration following infection was used as the reference for these calculations.

This review was registered on Prospero (CRD42022371944). Preferred Reporting Items for Systematic Review and Meta-Analyses (PRISMA) guidelines were followed when applicable in creation of this review.

## Results

**Fig 1:**
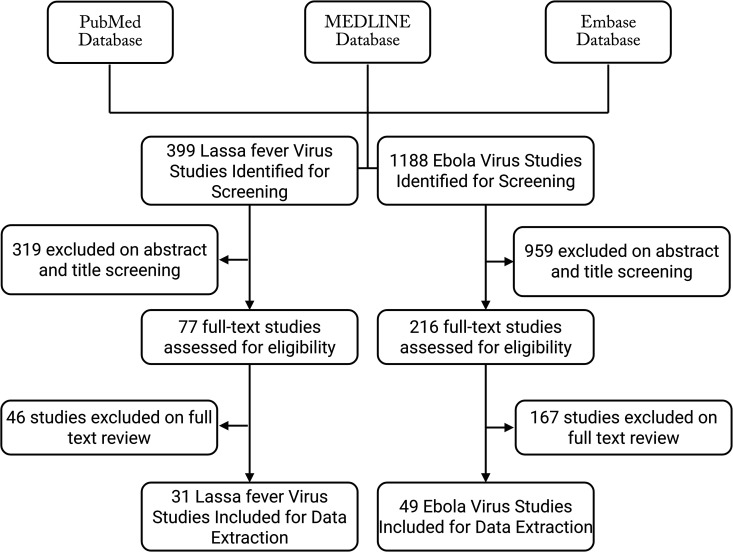
Review Methodology Search Results. Figure displays the articles obtained and excluded at each step in the review process for studies regarding immune system dysfunction in acute and convalescent cases of Ebola Virus Disease and Lassa fever.

Using this search strategy a total of 1,587 articles were identified and evaluated for inclusion. Specifically, 1,188 unique studies were screened related to the Ebola virus and 399 unique studies were identified regarding LF. Title and abstract screening found 959 and 319 studies were irrelevant for the Ebola and LF viruses, respectively. Of the 216 Ebola virus full text studies assessed, 49 articles were included in this analysis. Of the 77 LF virus full text studies reviewed, 31 were included in this analysis (Fig 1).

### Immune-inflammatory dysfunction following Ebola virus infection

#### Biomarkers and Cytokines following Ebola Infection.

In total, 23 articles included evaluations of biomarkers and cytokines following Ebola infection [21–44]. This includes 17 studies which reported measurements of serum biomarkers and cytokines among both survivors and fatal cases of infection.

#### Biomarkers and cytokines among Ebola survivors.

**Fig 2:**
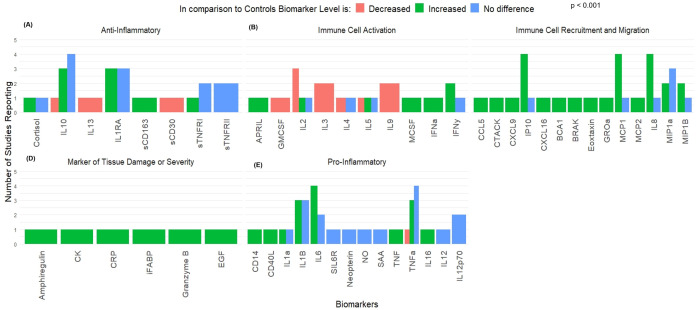
Changes to Serum Biomarkers Among EVD Survivors. Figure displays level of serum biomarkers in disease survivors in comparison to healthy controls. Biomarkers were grouped according to overall function as **(A)** Anti-Inflammatory mediators, **(B)** Immune cell activators, **(C)** Promoters of cell recruitment or migration, **(D)** Markers of tissue damage or severity of infection, or **(E)** Pro-Inflammatory mediators.

Thirteen articles reported information regarding 259 Ebola survivors in comparison to healthy controls [21–23,26,28–31,34,35,38,40,43]. The median number of days from disease onset to sample collection was 8 (Interquartile Range [IQR] = 6–11) days. In total, data was available for 52 unique cytokines or biomarkers. Biomarkers were grouped by overall function into the following categories: Anti-Inflammatory Molecules (n = 8 biomarkers), Promoters of Immune Cell Activation (n = 10 biomarkers), Promoters of Immune Cell Recruitment and Migration (n = 14), Markers of Tissue Damage (n = 6), or Pro-Inflammatory Molecules (n = 14, Fig 2). In total, there were 116 measurements of biomarker concentration. The majority of these (n = 91, 78%) occurring within 14 days from disease onset. Using Fisher’s Exact Test, a significant difference was observed in the frequency of observations of biomarkers and cytokines (p < 0.001). This was followed by multinomial logistic regression to determine which functional classes were significantly different and the direction and magnitude of the observed change in concentration. After assigning each change in direction a numerical value (Decreased = -1, No Difference = 0, Increased = 1), Promoters of Immune Cell Activation were found to be most neutral or centered on having no difference in concentration following infection (Mean = -0.142 ± 0.91) and were used as a reference group for regression modelling. This model found that Promoters of Immune Cell Recruitment were significantly more likely to be increased (Odds Ratio [OR] = 1.69, 95%CI = 1.06 – 2.72, p = 0.029). All other function classes were not significantly different from baseline. However, when controlling for observations made during the acute phase of disease (<= 14 days from disease onset, n = 91 observations), there were no significant findings among the observed change in biomarker concentration. Twenty-five observations were made in convalescence, with the majority (80%) collected one year following disease onset. During this time period, although all biomarker classes were observed to have significantly increased in concentration relative to controls, there was no difference found between functional groups. In cases where the numerical data regarding biomarker concentrations were not available, additional data was requested from study authors and included in the supplement where available (S1 Table).

#### Biomarkers and cytokines among fatal cases of Ebola.

**Fig 3:**
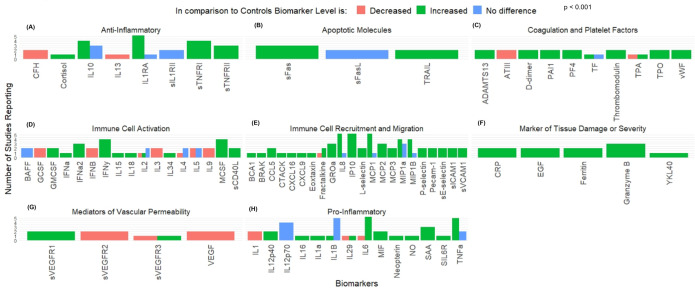
Changes to Serum Biomarker Level Following Fatal EVD Infection. Figure displays level of serum biomarkers in fatal Ebola infection in comparison to healthy controls. Biomarkers were grouped according to overall function as **(A)** Anti-Inflammatory mediators, **(B)** Promoters of Apoptosis, **(C)** Coagulation and Platelet Factors, **(D)** Immune cell activators, **(E)** Promoters of cell recruitment or migration, **(F)** Markers of tissue damage or severity of infection, **(G)** Mediators of Vascular Permeability, or **(H)** Pro-Inflammatory mediators.

Six articles reported data regarding serum biomarkers and cytokines among 271 cases of Fatal Ebola infection in comparison to healthy controls [22,23,28,37,38,43]. The median number of days from disease onset to sample collection was 5 (IQR = 5–8) days. Among this group, information was gathered concerning 83 unique cytokines or biomarkers. Biomarkers were grouped into the same functional classes as those used for survivors (Anti-Inflammatory Molecules [n = 8], Promoters of Immune Cell Activation [n = 17], Promoters of Immune Cell Recruitment and Migration [n = 22], Markers of Tissue Damage [n = 5], or Pro-Inflammatory Molecules [n = 14]) with additional functional classes for those not previously captured by prior classifications (Apoptotic Molecules [n = 3], Coagulation and Platelet Factors [n = 10], and Mediators of Vascular Permeability [n = 4], Fig 3). Fisher’s exact test noted that there was a significant difference among the observed frequencies in concentration. Using the same methodology as previous, the most neutral functional class was Promoters of Immune Cell Activation (Mean = 0.02 ± 0.88). Using this class as a reference group, logistic regression modelling was completed. This modelling demonstrated that Anti-Inflammatory (OR = 2.35, 95%CI = 1.95 – 2.84, p < 0.001), Pro-Apoptotic Molecules (OR = 1.36, 95%CI = 1.09 – 1.69, p = 0.007), Coagulation and Platelet Factors (OR = 9.86, 95%CI = 7.56 – 12.79, p < 0.001), Promoters of Immune Cell Recruitment (OR = 35.25, 95%CI = 26.31 – 47.22, p < 0.001) and Pro-Inflammatory Molecules (OR = 1.70, 95%CI = 1.44 – 1.9, p < 0.001) were significantly more likely to be increased during fatal Ebola infections. Comparisons were also made regarding the observed biomarker concentration between survivors and fatal infections. It was found that fatal infections were more likely to have increased concentrations of Promoters of Immune Cell Activation (50 vs 33%, p = 0.015) and Anti-Inflammatory Molecules (67 vs 38%, p = 0.024). There was also trend of increased concentration of Pro-Inflammatory Molecules (63 vs 45%, p = 0.091) and Promoters of Immune Cell Recruitment (89 vs 78%, p = 0.064). However, these findings were not significant. Measured cytokine level results were requested from study authors and are included where available (S2 Table).

#### Biomarkers and cytokines: mRNA analysis and uncontrolled studies.

Ten additional articles were found which reported data regarding biomarkers during the acute and convalescent phases of Ebola infection [24,25,27,32,33,36,39,41,42,44]. These articles included a variety of control groups and compared symptomatic to asymptomatic Ebola Virus Disease (EVD) survivors, critical vs non-critical clinical status and unique methodologies including measurements of gene expression, mRNA transcript levels, and other novel methods and were not comparable to the prior studies. Five of these articles estimated the levels of various biomarkers and cytokines through the measurement of mRNA, gene expression, and transcriptomic approaches [25,27,41,42,44]. Two studies found an increase in IL-6, IL-10, IFNγ, TNF, Fas, and FasL mRNA expression indicating increased immune cell activation, dysregulated inflammation, and apoptosis in fatal cases of disease compared to survivors [41,44]. One article compared severe and moderate infections, increased severity of infection was associated with increased coagulation, lymphocyte activity, and inflammation in comparison to more moderate cases of disease [33]. One study examined the levels of biomarkers using a novel methodology which noted increased levels of chemokines during the acute phase of infection [39]. Two articles compared EVD survivors with symptoms of Post-Ebola Syndrome (PES) to those without [24,36]. These articles demonstrate conflicting results as one notes significantly increased levels of IL-13 and decreased levels of MIP-1β and IL-1β, which contrasts with the other article which found no significant differences between groups. The last included article evaluated biomarker and cytokine differences between Sudan and Zaire Ebola Virus infections [32]. This study found increased levels of triglycerides, ferritin, soluble CD163, and soluble IL-2 receptors among survivors of Sudan Ebola Virus infections.

### Innate and adaptive immune cell populations.

In total, 11 articles included evaluations of innate and adaptive immune cell populations following Ebola infection [21,22,25,27,45–51]. Eight of these articles reported the frequency of innate and adaptive immune cell populations following infection.

#### Innate Cell Populations following Ebola Infection.

Five of these eight noted changes to immune cell frequencies among 52 Ebola Survivors. In total, there were 42 observations made regarding changes to cell population frequency [21,22,46,48,49]. Cell populations were grouped in to 3 major cell types which included Innate cells (n = 22 observations, 52%), T- (n = 13, 31%), and B- (n = 7, 17%) cells. Five articles reported changes to immune cell frequency among 75 fatal cases of Ebola. In total, there were 39 observations made across all major cell types (Innate [n = 7, 18%], T- [n = 28, 72%], and B- [n = 4, 10%] Immune Cells). The median number of days from infection to sample collection was 5.5 (IQR = 4-9.5) days.

**Fig 4:**
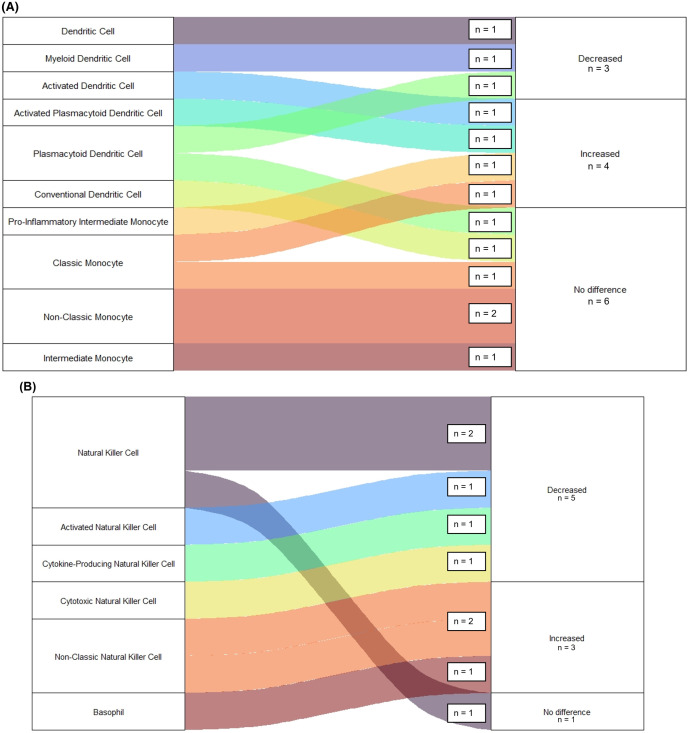
Observations of Changes to Innate Cell Frequencies Among EVD Survivors. Figure displays number of studies reporting changes to frequencies of Innate Immune Cell Populations following Ebola Infection in disease survivors. Panels display **(A)** Monocytes and dendritic cells and **(B)** Basophils and Natural Killer Cells. All studies reporting changes to Innate Immune Cell Frequencies were compared to a group of healthy uninfected controls.

Among survivors, 22 observations were made of Innate Immune Cell Populations [21,46,49]. This group includes: Dendritic and Monocytic Cells (Fig 4a) and Basophils and Natural Killer (NK) Cells (Fig 4b). Samples were collected a median time of 14 (IQR = 4–690) days following disease onset. Half of these observations were made using samples collected within 14 days of disease onset while the remaining samples were collected more than one year later. Among early observations of innate immune cells, 27% (n = 3) noted an increase while 36% (n = 4) noted a decrease in cell population frequency. The remaining observed no differences in comparison to healthy controls. No significant differences were found when comparing changes in population frequencies between cell types (p = 0.653) or comparing acute and convalescent samples (p = 0.216). However, convalescent samples did more commonly demonstrate a reduced frequency of innate cells (n = 6, 54%).

**Fig 5:**
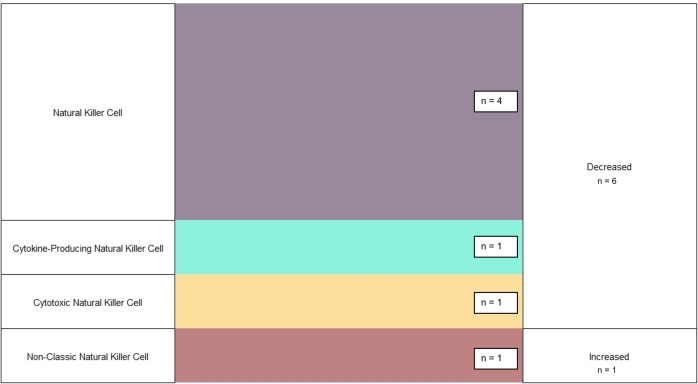
Observations of Changes to Innate Cell Frequencies Following Fatal EVD Infection. Figure displays number of studies reporting changes to frequencies of specific Innate Immune Cell Populations following Ebola Infection in fatal cases. All studies reporting changes to Innate Immune Cell Frequencies were compared to a group of healthy uninfected controls.

Seven observations were made regarding the Innate Immune Cell Population frequency (Fig 5) among Fatal EVD cases [27,49]. However, observations were only made of NK Cells. Similar to survivors, the most observations (n = 6, 86%) noted a reduction in NK Cell population frequency. In comparing survivors and fatal EVD, fatal cases were more likely to have reduced frequency of Innate Cells (p = 0.039). However, this finding was no longer significant after restricting to only NK Cell observations.

#### T-cell Populations following Ebola Infection.

**Fig 6:**
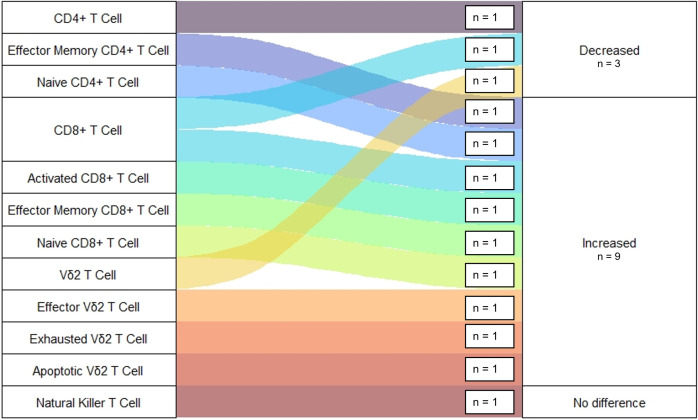
Observations of Changes to T-cell Frequencies Among EVD Survivors. Figure displays number of studies reporting changes to frequencies of specific T Cell Populations following Ebola Infection in disease survivors. All studies reporting changes to T Cell Frequencies among survivors were compared to a group of healthy uninfected controls.

Thirteen observations were made regarding T-cell Population frequencies among Ebola survivors (Fig 6) [21,22,46,48,49]. Of this cell type, the populations observed included; CD4^+^, CD8^+^, Vδ2^+^, and NK-like T-cell populations. Samples were collected a median of 15 (IQR = 11 – 37) days following disease onset. Only two observations were made of T-cell populations during the convalescent phase of illness more than 1 year following disease onset. Most of these observations (n = 8, 61%) noted an expansion in T-cell population frequency in comparison to healthy controls. No significant differences were found when comparing changes to frequency across T-cell Populations (p = 0.240) or any changes to frequency over time (p = 0.804).

**Fig 7:**
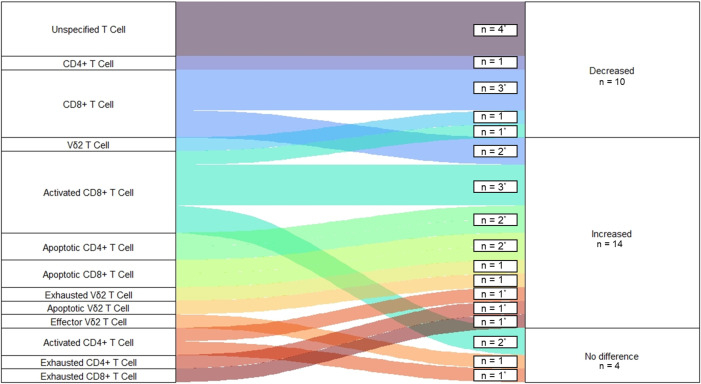
Observations of Changes to T-cell Frequencies Following Fatal EVD Infection. Figure displays number of studies reporting changes to frequencies of specific T Cell Populations following Ebola Infection in fatal cases. The majority studies reporting changes to T Cell Frequencies among EVD were compared to a group of healthy uninfected controls except in those which compared fatal cases to survivors. This difference is noted by an *.

In comparison, 28 observations were made of T-cell population frequencies during fatal EVD (Fig 7) [22,27,49,51]. This includes unspecified (n = 4, 14%), CD8^+^ (n = 14, 50%), CD4^+^ (n = 6, 21%) and Vδ2^+^ (n = 4, 14%) T-cell populations. These samples were collected a median of four (IQR = 4-9.5) days following disease onset. Most observations were made between fatal infections and survivors during the acute phase of disease. Similarly, most observations found an expansion in T-cell population frequency. Interestingly, nearly half of the expanded T-cell populations were noted to be exhausted or apoptotic. No significant differences were found when comparing across T-cell populations or changes in frequency over time (p = 0.371). Additionally, no significant differences were found between survivor and fatal EVD T-cell Population frequencies during the acute phase (p = 0.291).

#### B-cell populations following Ebola infection.

**Fig 8:**
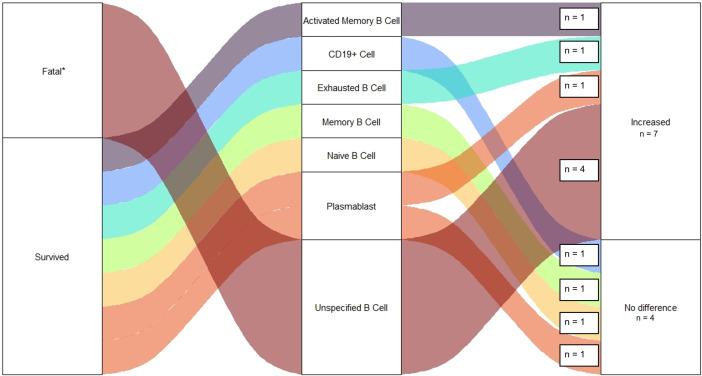
Observations of Changes to B-cell Frequencies Among EVD Cases. Figure displays number of studies reporting changes to frequencies of specific B Cell Populations following Ebola Infection in fatal cases and survivors. All studies reporting changes to B Cell Frequencies among survivors were compared to a group of healthy uninfected controls healthy. Except one study which examined changes to populations of B Cell frequencies among fatal cases compared to EVD survivors. This difference is noted by an *.

Seven observations were made regarding B-cell Population frequencies among Ebola survivors (Fig 8) [21,46]. This includes Activated Memory (n = 1, 17%), CD19^+^ (n = 1, 17%), Exhausted (n = 1, 17%), Memory (n = 1, 17%), Naïve (n = 1, 17%), and Plasmablast (n = 2, 29%) B-cells. Four of these observations were collected during the acute phase of EVD. Interestingly, of these early observations, one study had noted an increase in the population frequency of Plasmablasts in contrast to Naïve and Memory B-cells which had no difference relative to controls. However, more than year following disease onset, Activated Memory B-cells had increased in frequency. Only four observations were made of B-cells in fatal Ebola infections which found no difference in population frequency relative to healthy controls [27].

#### Immune cell population analysis: Receptor diversity, gene expression, and uncontrolled studies.

Three additional articles which were included but were incomparable to other studies evaluated population frequencies through indirect methodologies such as measurement of receptor diversity and expression of genes related to Innate and T-cell function, or included no control group [25,47,50]. These studies noted an expansion of proliferating T-cells and increased polyclonality in disease survivors [25,47]. Additionally two studies noted inversions of the CD4^+^/CD8^+^ among acute EVD survivors which returned to baseline at time of discharge [47,50].

### Antibody response and function

In total, 27 studies evaluated serum antibodies and antibody function following Ebola infection [21,26,30,34–36,40,42–44,47,52–67]. Nineteen of these included measurements of the serum antibody levels among survivors and fatal EVD or antibody prevalence among contacts of known cases or at risk populations (Table 1). Data was gathered regarding 622 EVD survivors (Table 1A) of which 507 (81%) had produced a specific antibody response [21,26,34,40,42–44,52–56,60–64,67]. Of 373 survivors, 96% (n = 349) were found to have neutralizing activity. Sixty-four survivors were evaluated during the acute phase of illness. Of these, 51% (n = 33) had developed a specific antibody response which varied from 5-92%. This is in comparison to the 33 cases of fatal EVD (Table 1B). Among these cases there was a total antibody prevalence of 36% (n = 12) but ranged from 0-82% [40,42,43]. Although there was a trend of increased antibody prevalence among acute survivors of EVD this finding was not significant (p = 0.199). Among 4626 contacts and other at risk individuals approximately 17% were serologically positive for Ebola antibodies (Table 1C) [60,61,65]. Among 107 of these individuals only 3 were found to be capable of neutralizing the virus [61].

**Table 1:** Antibody response following Ebola infection.

First Author	Study Year	Country	EBV Species	Sample Size	Sample Type	Median Age (Years)	Sex (% Male)	Days Since Onset	Reactive to EBV Antigens N (%)	Neutralizing Activity N (%)	Control Group (N)
**(A)**											
Baize [44]	1999	Gabon	Zaire	20	Survivor	NG	NG	3	1 (5)	NG	No
LeRoy [34]	2000	Gabon	Zaire	24	Survivor	NG	NG	16.5	11 (46)	NG	No
Baize [43]	2002	Gabon	Zaire	8	Survivor	23	NG	2	8 (100)	NG	Yes (12)
Gupta [40]	2012	Uganda	BDBG	12	Survivor	NG	NG	7.5	11 (92)	NG	Yes (10)
Gupta [40]	2012	Uganda	BDBG	21	Survivor	NG	NG	49.5	21 (100)	NG	Yes (10)
Sobarzo [56]	2012	Uganda	Sudan	78	Survivor	NG	NG	NG	23 (29)	NG	Yes (100)
Sobarzo [26]	2015	Uganda	Sudan	5	Survivor	NG	NG	NG	5 (100)	5 (100)	Yes (3)
Sobarzo [55]	2016	Uganda	Sudan	15	Survivor	39	33	4380	12 (80)	6 (40)	Yes (5)
Bramble [67]	2018	Republic of Congo	BDBG	15	Survivor	NG	NG	365	15 (100)	4 (80)*	No
Herrera [60]	2018	Nigeria	Zaire	3	Survivor	34	63	150	3 (100)	NG	Yes (6)
Sobarzo [54]	2019	Uganda	Sudan	15	Survivor	41	33	5475	9 (60)	6 (40)	Yes (3)
Williamson [52]	2019	NG	Zaire	4	Survivor	NG	NG	30	4 (100)	1 (25)	No
Halfman [61]	2019	Sierra Leone	Zaire	214	Survivor	NG	NG	705	209 (97)	208 (97)	Yes (38)
Colavita [42]	2019	Sierra Leone	Zaire	13	Survivor	NG	NG	7.5	4 (31)	NG	No
Davis [64]	2019	NG	Zaire	4	Survivor	NG	NG	NG	4 (100)	NG	No
Wiedeman [21]	2020	Guinea	Zaire	34	Survivor	30	NG	690	34 (100)	NG	Yes (39)
Dean [63]	2020	NG	Zaire	6	Survivor	NG	NG	168	6 (100)	6 (100)	No
Thom [53]	2020	Guinea	Zaire	117	Survivor	NG	NG	255	113 (96)	113 (96)	Yes (23)
Gunn [62]	2020	Sierra Leone	Zaire	14	Survivor	NG	NG	180	14 (100)	NG	Yes (3)
**(B)**											
**First Author**	**Study Year**	**Country**	**EBV Species**	**Sample Size**	**Sample Type**	**Median Age (Years)**	**Sex** **(% Male)**	**Days Since Onset**	**Reactive to EBV Antigens** **N (%)**	**Neutralizing Activity** **N (%)**	**Control Group** **(N)**
Baize [43]	2002	Gabon	Zaire	9	Fatal Disease	23	NG	9	0	NG	Yes (12)
Gupta [40]	2012	Uganda	BDBG	11	Fatal Disease	NG	NG	7	9 (82)	NG	Yes (10)
Colavita [42]	2019	Sierra Leone	Zaire	13	Fatal Disease	NG	NG	7.5	3 (23)	NG	No
**(C)**											
**First Author**	**Study Year**	**Country**	**EBV Species**	**Sample Size**	**Sample Type**	**Median Age (Years)**	**Sex** **(% Male)**	**Days Since Contact**	**Reactive to EBV Antigens** **N (%)**	**Neutralizing Activity** **N (%)**	**Control Group** **(N)**
Herrera [60]	2018	Nigeria	Zaire	10	Disease Contacts	34	63	150	2 (20)	NG	Yes (6)
Halfman [61]	2020	Sierra Leone	Zaire	267	Disease Contacts	NG	NG	705	107 (40)	7 (3)	Yes (38)
Becquart [65]	2010	Gabon	Zaire	4349	Prevalence Study Population	NG	48.4	NG	667 (15)	NG	No

Tables display the sample sizes and functional properties among **(A)** Ebola Survivors, **(B)** Fatal Ebola Infection, and **(C)** known disease contacts and population sampling. Abbreviations: NG, Not Given; BDBG, Bundibugyo. * Indicates where specific testing only occurred on a subset of samples (n = 5).

#### Antibody analysis: Kinetics and function.

Ten studies included evaluations of Ebola antibodies and antibody function that were performed using novel methods or control groups [30,35,36,46,47,57–59,64,66]. These results showed that EVD survivors develop a peak IgG response approximately two weeks following symptom onset. Most of these antibodies are specific for Ebola glycoprotein and are capable of viral neutralization up to ten years following viral exposure. Additionally, the majority of antibodies are polyfunctional and capable of antibody-dependent cellular phagocytosis, complement deposition, and natural killer cell activation more than one year following infection.

### Cellular function following Ebola antigen stimulation

Seven studies included evaluations of cellular function following Ebola antigen stimulation. These experiments were conducted on samples collected from 247 Ebola survivors and 13 cases of acute infection [21,36,53,54,60,68,69]. A positive response was defined as an increased expression of IFNγ (n = 7, 100% of studies), TNFα (n = 4, 57% of studies), CD107a, MIP1-β, or IL-2 (1, 14% of studies). These studies found that both fatal cases and survivors of Ebola generated an increased response in comparison to healthy controls. In addition, one study which compared survivors with PES to survivors without sequelae found had an increased CD8^+^ T-cell and CD4^+^ T-cell expression of IFNγ and TNFα following stimulation with Ebola antigens [36].

### Immune-Inflammatory Dysfunction Following Lassa Fever Virus Infection

#### Biomarkers and cytokines following Lassa fever infection.

Eight articles evaluated biomarkers and cytokines following LF infection. In total, information was collected regarding 129 LF cases including 48 (37%) disease survivors and 81 (73%) fatal infections [14,15,70–75].

#### Biomarkers and cytokines among Lassa fever survivors.

**Fig 9:**
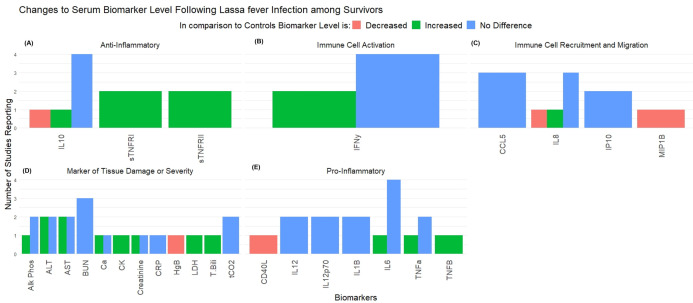
Changes to Serum Biomarker Level among Lassa fever Survivors. Figure displays level of serum biomarkers in disease survivors in comparison to healthy controls. Biomarkers were grouped according to overall function as **(A)** Anti-Inflammatory mediators, **(B)** Immune cell activators, **(C)** Promoters of cell recruitment or migration, **(D)** Markers of tissue damage or severity of infection, or **(E)** Pro-Inflammatory mediators.

Among the 48 survivors, 4 articles reported information on 27 unique biomarkers (Fig 9) [14,15,71,73]. Biomarkers were grouped according to overall function, similar to EVD survivors as; Anti-Inflammatory (n = 3 biomarkers), Promoters of Immune Cell Activation (n = 1), Promoters of Immune Cell Recruitment and Migration (n = 4), Markers of Tissue Damage or Disease Severity (n = 12), or Pro-Inflammatory Molecules (n = 7). Using the same methodology as previously described, Pro-Inflammatory Molecules were found to be the most neutral class (Mean = 0.125 ± 0.5) and were used as reference category for logistic regression. This modelling found no significant differences in concentration among the different functional classes of biomarkers. Only one study evaluated biomarkers during convalescence [73]. This study found that among 16 LF survivors 56 days following symptom onset that all biomarkers had returned to baseline and had no difference from healthy controls.

#### Biomarkers and cytokines among Fatal Lassa fever cases.

**Fig 10:**
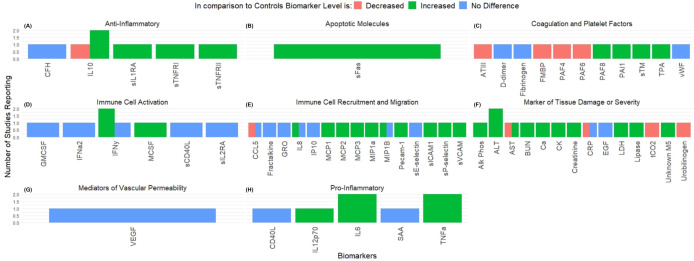
Changes to Serum Biomarkers following Fatal Lassa fever Infection. Figure displays level of serum biomarkers in fatal Lassa fever infection in comparison to healthy controls. Biomarkers were grouped according to overall function as **(A)** Anti-Inflammatory mediators, **(B)** Promoters of Apoptosis, **(C)** Coagulation and Platelet Factors, **(D)** Immune cell activators, **(E)** Promoters of cell recruitment or migration, **(F)** Markers of tissue damage or severity of infection, **(G)** Mediators of Vascular Permeability, or **(H)** Pro-Inflammatory mediators. Mean values of reported biomarkers was determined when available and displayed above each column. Several studies did not report measurements and are labelled as Not Given (NG). All mean values are reported as pg/ml except those noted by * to indicate ng/ml and ^†^ to indicate µg/ml.

Four of the included articles evaluated 57 unique biomarkers and cytokines among 81 fatal infections (Fig 10) [14,70,72,73]. Biomarkers and cytokines measured in fatal cases of infection were classified by overall function into the following categories; Anti-Inflammatory (n = 5 biomarkers), Apoptotic (n = 1), Coagulation and Platelet Factors (n = 11), Immune Cell Activation (n = 6), Immune Cell Recruitment and Migration (n = 14), Markers of Tissue Damage or Severity (n = 14), Mediators of Vascular Permeability (n = 1), and Pro-Inflammatory (n = 5) Molecules. There were no significant differences found between biomarkers following infection (p = 0.443). Additional comparisons were made between survivors and fatal cases of infection. However no significant differences were found (p = 0.123).

#### Biomarkers and cytokines analysis: Markers of platelet function and coagulation.

An additional study evaluated platelet dysfunction and coagulation markers among both survivors and fatal cases of LF using both febrile and healthy control groups during the acute phase of illness [74]. This study found significant increases in the level of specific markers of coagulation and fibrinolysis. In addition, it was noted that several of these markers were associated with increasing levels of viremia. The last remaining study evaluated the levels of viremia and AST at admission, this article demonstrated that elevations in this biomarker were significantly associated with fatal outcomes [75].

### Innate and adaptive immune cell populations

Only one study evaluated immune cell populations following LF infection [76]. This study found that during acute symptomatic LF infection, there is an expansion of the activated CD8^+^ T-cells defined as CD38^+^/CD8^+^ cells above the normal reference range.

### Antibody response and function

Twenty-three articles included evaluations of LF virus specific antibodies following infection [14,45,71,73,75,77–94]. This included studies regarding 471 LF survivors during the convalescent phase of illness and additional evaluations of 673 cases of the acute phase of disease for which the outcome was not reported.

#### Antibody response and function among Lassa fever survivors.

Ten studies were included that evaluated 471 LF survivors [14,45,71,73,77,84,85,88,91,92]. Five articles (n = 63, 13%) included evaluations that were conducted during the acute phase of illness while the remaining five studies had used samples from survivors (n = 408, 87%) drawn during convalescence (Table 2A). Convalescent samples were drawn a median of 75 (IQR = 19 – 220) days following LF disease onset. Among this group of convalescent survivors, the majority (351, 86%) had generated an antibody response. These convalescent samples were most often assessed for a combination of IgG and IgM. However two articles did not specify antibody class. In comparison nearly all acute survivor samples were serologically positive (n = 62, 98%) at the sample of collection. Follow up samples collected from acute cases of LF demonstrated a stability in the IgM response for several years following initial infection [73,84,85,91]. IgG had also become detectable in acute samples from LF survivors early in the disease course, and remained positive several months later [71,73,85]. Notably, IgG responses had still been detected in survivors more than 40 years following the initial infection [77].

**Table 2:** Antibody response following Lassa fever infection.

First Author	Study Year	Location	Sample Size	Sample Type	Median Age		Sex % Male	Time Since Symptom Onset (Days)	Cases Reactive N (%)	Controls Reactive N (%)		Most Reactive Antigen	Mean IgG Antibody Titer Reciprocal	Cases with Neutralizing Acttivity N (%)	
**(A)**															
Jahrling [84]	1985	Liberia	26	Survivor	NG		NG	6	26 (100%)	NA		NG	NG	7^†^	
														20^†^	
														26^†^	
ter Meulen [45]	2000	Guinea	12	Survivor	27		75	NG	NG	NA		NG	1280	NG	
Schmitz [14]	2002	Ivory Coast	1	Survivor	22		0	6	0	NA		NA	NA	NA	
Macher [88]	2006	Sierra Leone	1	Survivor	26		0	60	1 (100%)	NA		NG	256	NG	
Macher [88]	2006	Sierra Leone	1	Survivor	43		0	90	1 (100%)	NA		NG	64	NG	
Branco [73]	2011	Sierra Leone	34	Survivor	NG		NG	3	34 (100%)	63 (62.4%)		NG	NG	NG	
Branco [71]	2011	Sierra Leone	1	Survivor	22		0	10	1 (100)	NA		NP	NG	NG	
Grove [85]	2011	Sierra Leone	1	Survivor	8		100	2	1 (100%)	NA		NP	NG	NG	
Bond [77]	2013	Nigeria	2	Survivor	NG		50	14600	2 (100)	NA		GP	8100	NG	
Shaibu [91]	2021	Nigeria	22	Survivor	46.5		42.5	NG	16 (73%)	123 (58%)		NG	NG	NG	
Ugwu [92]	2022	Nigeria	370	Survivor	32.59		54.5	NG	285 (77%)	109 (64%)		NP	NG	24 (40%)	
**(B)**															
**First Author**	**Study Year**	**Location**	**Sample Size**	**Sample Type**	**Median Age**	**Sex** **% Male**		**Time Since Onset (Days)**	**Cases Reactive** **N (%)**	**Controls Reactive** **N (%)**		**Most Reactive Antigen**	**Mean IgG Antibody Titer Reciprocal**		**Cases with Neutralizing Acttivity** **N (%)**
Jahrling [83]	1985	Liberia	1	Acute	NG	NG		5	1 (100%)	NA		NG	<10		NG
Jahrling [83]	1985	Liberia	1	Acute	NG	NG		5	1 (100%)	NA		NG	10		NG
Jahrling [83]	1985	Liberia	1	Acute	NG	NG		5	1 (100%)	NA		NG	<10		NG
Webb [93]	1986	Sierra Leone	23	Acute	“Children”	NG		13	21	NG		NG	400		NG
Johnson [75]	1987	Sierra Leone	128	Acute	NG	NG		3	70 (55%)	NA		NG	NG		NG
Johnson [75]	1987	Sierra Leone	136	Acute	NG	NG		9.5	106 (78%)	NA		NG	NG		NG
Johnson [75]	1987	Sierra Leone	71	Acute	NG	NG		15.5	63 (89%)	NA		NG	NG		NG
Bausch [79]	2001	Guinea	311	Acute	29	46		9	48 (15)	NA		NG	400		NG
Schmitz [14]	2002	Nigeria	1	Acute Fatal	48	100		9	1 (100%)	NA		NG	256		NG
**First Author**	**Study Year**	**Location**	**Sample Size**	**Sample Type**	**Median Age**	**Sex** **% Male**		**Time Since Onset (Days)**	**Cases Reactive** **N (%)**		**Controls Reactive** **N (%)**	**Most Reactive Antigen**	**Mean IgG Antibody Titer Reciprocal**		**Cases with Neutralizing Activity** **N (%)**
Fabiyi [82]	1975	Sierra Leone	170	Prevalence HCW	NG	NG		NA	12 (7%)		NA	NG	8		NG
Bloch [78]	1978	Liberia	165	Prevalence HCW	20-49	31		NA	6 (3.7%)		NA	NG	60		NG
Frame [81]	1984	Liberia	176	Suspected Febrile	NG	NG		0	21 (12%)		NA	NG	348*		NG
Yalley-Ogunro [94]	1984	Liberia	1848	Prevalence	NG	NG		NA	98 (5%)		NA	NG	>8*		NG
Akoua-Koffi [80]	2006	West Africa	163	Prevalence	NG	100		NA	88 (9%)		NA	NG	NG		NG
Shaffer [90]	2014	Sierra Leone	1740	Suspected Acute	1740	NG		0	407 (23%)		NA	NG	NG		NG
Kraft [87]	2020	US	44	Exposed	NG	NG		NA	0 (0%)		NA	Whole LV	<10		NG
Grant [86]	2023	Sierra Leone	10642	Prevalence	NG	51		NA	1701 (16%)		NA	NG	NG		NG

Tables display the sample sizes and functional properties of antibodies among **(A)** Lassa fever Survivors, **(B)** Acute Lassa fever Infections, and **(C)** exposed and at risk populations, †Tested individuals at 3 different time points (3, 6, and 8 months or more). Abbreviations NG, Not Given; NA, Not Applicable

#### Antibody response and function during acute Cases of Lassa fever.

Five studies evaluated the antibody response among 673 acute cases for which the disease outcome is unknown (Table 2B) [14,75,79,83,93]. Among this grouping, 315 (47%) individuals were found to mount an antibody response a median of five (IQR = 5 – 9.5) days following disease onset. An additional eight studies evaluated antibody response among suspected cases (two studies) presenting to a hospital site, exposed hospital staff (one study), and conducted prevalence (five studies) studies in endemic areas (Table 2C) [78,80–82,86,87,90,94]. Among the two studies which evaluated suspected febrile individuals, LF was thought to be the causative pathogen in 12% and 26% of cases presenting to hospital sites in Liberia and Sierra Leone, respectively [81,90]. The difference in LF incidence between these two studies is likely explained by the seasonal variation in LF, as one of these only monitored for cases during the rainy season when incidence is thought to be low [84]. A third study monitored for seroconversion among hospital workers caring for an imported case of LF in the United States [87]. None of the health care workers were found to be serologically positive throughout the course of the study. The five remaining prevalence studies were conducted among 12,825 individuals [78,80,82,86,94]. Of this group 1859 (14%) had produced antibodies specific to the LF virus.

### Cellular Function following Antigen Stimulation

Five studies evaluated the cellular response following stimulation with LF antigens or during the acute phase of illness [76,92,95–97]. In total, data was collected from 252 individuals. Samples were stimulated with either NP and GP peptide pools or recombinant Vesicular Stomatitis Virus (VSV) capable of producing LF NP, GP, or SSP proteins. These studies found that the majority of LF survivors (132, 52%) were capable of mounting a cellular response to antigens. Most studies found that cells mounted the strongest response to NP antigens. Of those individuals that did not respond to stimulation, one study found that extended stimulations of ten days elicited a response in the majority (67%) of individuals [95]. In addition, it was noted that individuals that did not respond to initial stimulations were more than ten years removed from LF infection. One study examined the T-cell responses during acute infection and noted that although there was an expansion of activated T-cells, that only a minority of there were specific to LF [76]. It was hypothesized that an increased frequency of non-specific cell may limit the T-cell response to LF leading to worse disease outcomes [76].

## Discussion

The results of this review demonstrate that during the acute phases of disease both survivors and fatal cases of EVD exhibit increased concentrations of immune cell recruitment and migration molecules, particularly IP-10, MCP-1, and IL-8 which were commonly found to be elevated within 14 days of infection regardless of disease outcome. These molecules are specifically associated with recruitment of CD8^+^ T-cells, NK cells, dendritic cells, monocytes and macrophages to inflamed tissue. This is in congruence with the other findings of this study in survivors which demonstrated an expansion of CD8^+^ T-cells, monocytic cells, and activated and plasmacytoid dendritic cells. Although, it is difficult to determine their role in survival given a paucity of data regarding cell types in fatal disease. Additionally, the selective loss of most NK cell subsets, except for non-classical cells may suggest that NK cells play a lesser role in promoting survival. Although both survivors and fatal cases demonstrated expansion of the CD8^+^ T-cell compartment it is worth noting that nearly half of the expanded population among fatal cases carried markers of apoptosis or exhaustion which may suggest an ineffective response. Although these specific cell phenotypes were not observed among disease survivors, the heightened response to Ebola antigens up to two years following infection suggests the possibility of a more robust and longitudinal cytotoxic response. In regard to the humoral response, this review found that over 96% of disease survivors were found capable of generating EVD specific responses. Additionally, over 50% of these individuals had EVD specific antibodies during the acute phase of illness compared to only 36% of fatal cases. In summary, the loss of NK cells and expansion of exhausted and apoptotic lymphocytes, a limited humoral response, and increased concentration of molecules associated with apoptosis, coagulation and both pro- and anti-inflammatory effects characterize fatal cases of disease. These findings likely all contribute to failure to control the virus and eventual hemodynamic collapse, multi-organ failure and death secondary to a clinical picture consistent with distributive shock [98–103]. This in contrast to survivors which demonstrated a robust cellular response which is noted by a relative increase in innate and adaptive immune cell populations which is maintained during periods of convalescence. However, these results must be interpreted with caution as observations across disease outcomes were heterogenous with disease survivors lacking studies which measured markers of coagulation and platelet function, mediators of vascular permeability and cellular studies examining exhausted or apoptotic lymphocytes, or the relation of these findings to development of PES. Furthermore, fatal cases of disease were also found to have a limited data set specifically examining more diverse populations of innate cells and B-cell phenotypes.

The known evidence of Ebola pathogenesis is congruent with these findings. Upon infection the virus invades dendritic and other monocytic cells [104]. These early targets of infection are the primary sites for viral replication and aid in viral dissemination [105,106]. This likely explains why these cell types were more likely to have expanded or have no difference in population frequency following infection. In contrast NK, CD4^+^, and CD8^+^ which are classically associated with anti-viral responses, were more likely to be reduced in frequency or carry markers of exhaustion or apoptosis. This may be secondary to viral proteins 24 and 35 and surface glycoproteins which are known to impair the type I interferon response and are directly toxic to these cell types through caspase dependent and independent mechanisms [107–112]. Additionally, the surface glycoprotein has been shown to induce massive chemokines and cell attractants which aid in further dissemination and is demonstrated by the pronounced increase of molecules of recruitment shown by this review [113,114]. This has been demonstrated within hours of viral exposure leading to further attraction of target cells and viral replication [114,115]. As disease progresses, this increased T-cell apoptosis in fatal EVD is likely responsible for the impairment in humoral responses relative to survivors due to a lack of CD4^+^ dependent activation [22,98,116–118].

Less is known regarding the pathogenesis of LF. However, the findings of this review demonstrate a severe dysfunction in normal immuno-inflammatory pathways. Although studies have demonstrated fatal disease outcomes are associated with increased levels of viremia, the exact mechanisms through which the virus causes disease is uncertain. Its notable, however, that changes to specific biomarkers indicative of pro-inflammatory states, and immune cell activation have been observed during convalescence which indicates an ongoing immuno-inflammatory process following the acute phase of disease. These findings are consistent with hypotheses of Lassa pathogenesis: an inability to control replication of the LF virus leads to a T-cell driven inflammatory cascade ultimately causing distributive shock and multi-organ failure [13,119,120].

Currently acute treatment for both diseases is focused on supportive care. Given the hemorrhagic manifestations, maintaining appropriate hemodynamics and oxygen delivery is of upmost importance to prevent cardiovascular collapse and downstream organ damage. Electrolyte imbalances and renal injury are common and have been reported in both viruses complicating the delivery of supportive care and fluid management [121,122]. Although there are no specific treatments, there are several monoclonal antibody cocktails and other antiviral medications under investigation [122,123].

Following survival of Ebola or LF, nearly all individuals develop post viral syndromes [5,8,124]. In Post-Ebola syndrome, large cohort studies have reported symptoms of cough, dyspnea, cardiac manifestations, musculoskeletal pain, abdominal pain, and vision deficits [5,6,125]. The exact mechanisms of pathogenesis are unclear. However, it has been hypothesized that viral persistence may lead to direct viral damage and a chronic inflammatory states [126]. Lassa fever survivors also develop post-viral sequalae, most notably hearing loss or deafness [7]. The pathogenesis of LF sequelae is poorly understood, but evidence from non-human primates has demonstrated pathology similar to an ANCA-associated vasculitis [127]. The results of this review demonstrate a persistent disruption in the immuno-inflammatory homeostasis following survival. Although it is unclear how these immunologic changes and post-viral syndromes are related it is worth noting that other more common viral sequelae have been associated with underlying immune dysregulation [128–135].

In conclusion, the results of this review demonstrate a massive dysfunction in immuno-inflammatory homeostasis due to infection with the hemorrhagic fever viruses, Ebola and LF. This dysfunction appears to begin during the acute phase of infection and may persist for decades in survivors of both diseases. These alterations in function may be related to the development of post-viral syndromes and convalescent symptoms. However, current evidence is lacking. The utility of this and other similar reviews are hindered by the lack of standardized and numerical data reported in primary studies. Additional key limitations of this study include the lack of detailed information regarding the clinical status of hemorrhagic fever patients and the heterogeneity of assessed cell subsets and biomarkers across disease outcomes. Future studies of acute cases of hemorrhagic fever would do well to include more robust assessments of immune-inflammatory dysfunction and the relationship of these findings to Post-viral syndromes.

## Supporting information

S1 Table Mean reported biomarker levels among Ebola survivors. Table displays the mean reported values for biomarkers measured among Ebola Survivors. Majority of studies did not report measured values. Table displays measurements for all biomarkers for which data was available. All study authors were contacted regarding raw data requests. Standard deviations were calculated where multiple studies reported measured values. All measured values reported as pg/ml unless noted by * to indicate ng/ml
(DOCX)

S2 Table Mean biomarker levels among fatal EVD Cases. Table displays the mean value reported for biomarkers among Ebola infection. All study authors were contacted regarding raw data requests. Standard deviations were calculated where multiple studies reported measured values. All measured values reported as pg/msCD40Ll unless noted by * to indicate ng/ml
(DOCX)

S1 Data 2020 PRISMA systematic review checklist. (DOCX)
