# Supplementary material for: A systematic review of the immuno-inflammatory dysfunction secondary to viral hemorrhagic fevers; Ebola and Lassa fever
Source: PLoS Negl Trop Dis. 2025 Jun 25;19(6):e0013230. doi: 10.1371/journal.pntd.0013230 (PMC12240328; doi:10.1371/journal.pntd.0013230)
Supplement: S1 Table — Table displays the mean reported values for biomarkers measured among Ebola Survivors. Majority of studies did not report measured values. Table displays measurements for all biomarkers for which data was available. All study authors were contacted regarding raw data requests. Standard deviations were calculated where multiple studies reported measured values. All measured values reported as pg/ml unless noted by * to indicate ng/ml (DOCX) [file pntd.0013230.s001.docx]

**S1 Table.** **Mean Reported Biomarker Levels Among Ebola Survivors**

| **Biomarker** | **Number of Studies Included** | **Number of Studies Reporting Measurements** | **Value in Ebola Cases**  **Mean (SD)** | **Value in Ebola Controls**  **Mean (SD)** |
| --- | --- | --- | --- | --- |
| Amphiregulin | 1 | 1 | 8.4 | 5.5 |
| Cortisol* | 2 | 2 | 104.0 (147) | 460.0 (650) |
| IFNa | 1 | 1 | 42.0 | 16.0 |
| IFNy | 3 | 1 | 49.0 | 43.0 |
| IL10 | 8 | 2 | 97.5 (74) | 25.0 (35) |
| IL1RA* | 6 | 3 | 146.3 (249) | 110.4 (188) |
| IL6 | 6 | 1 | 229.0 | 109.0 |
| IL8 | 5 | 1 | 33.0 | 14.0 |
| MIP1a* | 5 | 1 | 0.3 | 0.2 |
| MIP1B* | 3 | 1 | 0.5 | 0.9 |
| Neopterin* | 1 | 1 | 14.8 | 2.2 |
| NO | 1 | 1 | 136.0 | 20.0 |
| sIL6R* | 1 | 1 | 51.8 | 20.0 |
| sTNFRI* | 3 | 1 | 2.3 | 1.8 |
| sTNFRII | 2 | 1 | 9.9 | 5.0 |
| TNFa | 8 | 2 | 64.8 (68) | 84.8 (102) |

Table displays the mean reported values for biomarkers measured among Ebola Survivors. Majority of studies did not report measured values. Table displays measurements for all biomarkers for which data was available. All study authors were contacted regarding raw data requests. Standard deviations were calculated where multiple studies reported measured values. All measured values reported as pg/ml unless noted by * to indicate ng/ml
