# Supplementary material for: A systematic review of the immuno-inflammatory dysfunction secondary to viral hemorrhagic fevers; Ebola and Lassa fever
Source: PLoS Negl Trop Dis. 2025 Jun 25;19(6):e0013230. doi: 10.1371/journal.pntd.0013230 (PMC12240328; doi:10.1371/journal.pntd.0013230)
Supplement: S2 Table — Table displays the mean value reported for biomarkers among Ebola infection. All study authors were contacted regarding raw data requests. Standard deviations were calculated where multiple studies reported measured values. All measured values reported as pg/msCD40Ll unless noted by * to indicate ng/ml (DOCX) [file pntd.0013230.s002.docx]

**S2 Table. Mean Biomarker Levels Among Fatal EVD Cases**

| **Biomarker** | **Number of Studies Included** | **Number of Studies Reporting Measurements** | **Value in Ebola Cases**  **Mean (SD)** | **Value in Ebola Controls**  **Mean (SD)** |
| --- | --- | --- | --- | --- |
| ADAMSTS13^†^ | 2 | 2 | 1.3 (1.5) | 1 (0) |
| ATIII^†^ | 2 | 2 | 226 (358) | 3092 (3578) |
| CCL5 | 2 | 2 | 332 (51) | 58 (0) |
| CFH^†^ | 2 | 2 | 269 (0) | 629 (0) |
| Cortisol* | 1 | 1 | 392 | 92 |
| CRP^†^ | 2 | 2 | 79 (33) | 2 (0) |
| D-dimer^†^ | 2 | 2 | 48 (13) | 2 (0) |
| EGF | 2 | 2 | 89 (25) | 44 (0) |
| Ferritin^†^ | 2 | 2 | 3 (0) | 0 (0) |
| Fractalkine | 3 | 2 | 136 (127) | 63 (0) |
| GCSF | 2 | 2 | 17 (18) | 70 (0) |
| GMCSF | 2 | 2 | 6 (4) | 2 (0) |
| Granzyme B* | 3 | 2 | 7 (0) | 0 (0) |
| GROa* | 3 | 2 | 10 (47) | 1 (0) |
| IFNa | 1 | 1 | 221 | 16 |
| IFNa2 | 3 | 2 | 107 (0) | 10 (0) |
| IFNB | 2 | 2 | 22 (1) | 65 (0) |
| IFNy | 4 | 3 | 161 (52) | 18 (22) |
| IL1 | 2 | 2 | 0 (0) | 6 (0) |
| IL10 | 7 | 3 | 320 (204) | 17 (28) |
| IL12p40 | 2 | 2 | 19 (0) | 1 (0) |
| IL1RA | 7 | 3 | 4636 (7678) | 534 (923) |
| IL2 | 4 | 1 | 137 | 82 |
| IL29 | 2 | 2 | 408 (25) | 405 (0) |
| IL6 | 8 | 4 | 64 (85) | 28 (54) |
| IL8 | 7 | 2 | 200 (163) | 3 (0) |
| IP10 | 7 | 2 | 42 (0) | 1 (0) |

**S2 Table Continued. Mean Biomarker Levels in Fatal EVD Cases**

| **Biomarker** | **Number of Studies Included** | **Number of Studies Reporting Measurements** | **Value in Ebola Cases**  **Mean (SD)** | **Value in Ebola Controls**  **Mean (SD)** |
| --- | --- | --- | --- | --- |
| L-selectin^†^ | 2 | 2 | 1 (0) | 0 (0) |
| MCP1* | 7 | 2 | 1 (1) | 0 (0) |
| MCP2 | 3 | 1 | 319 | 15 |
| MCP3 | 2 | 2 | 55 (28) | 1 (0) |
| MCSF* | 4 | 2 | 59 (70) | 0 (0) |
| MIP1a | 7 | 3 | 174 (200) | 75 (108) |
| MIP1B | 5 | 3 | 384 (449) | 315 (506) |
| Neopterin* | 1 | 1 | 137 | 2 |
| NO | 1 | 1 | 160 | 60 |
| PAI1* | 2 | 2 | 374 (217) | 51 (0) |
| Pecam-1* | 2 | 2 | 4 (1) | 2 (0) |
| PF4^†^ | 2 | 2 | 7 (0) | 2 (0) |
| P-selectin | 2 | 2 | 2 (0) | 0 (0) |
| SAA^†^ | 3 | 3 | 36 (31) | 5 (4) |
| sCD40L* | 2 | 2 | 60 (1) | 1 (0) |
| sE-selectin* | 2 | 2 | 72 (12) | 44 (0) |
| sFas* | 3 | 2 | 35 (4) | 21 (0) |
| sICAM1* | 2 | 2 | 549 (158) | 125 (0) |
| SIL6R* | 1 | 1 | 43 | 20 |
| sTNFRI | 4 | 3 | 8159 (8675) | 1080 (623) |
| sTNFRII* | 3 | 3 | 32 (11) | 4 (1) |
| sVCAM1^†^ | 2 | 2 | 24 (4) | 1 (0) |
| sVEGFR1* | 2 | 2 | 15 (13) | 1 (0) |
| sVEGFR2* | 2 | 2 | 12 (1) | 16 (0) |
| sVEGFR3 | 2 | 2 | 761 (320) | 565 (0) |
| TF | 2 | 1 | 300 | 100 |
| Thrombomodulin | 2 | 2 | 24 (13) | 8 (0) |
| TNFa | 7 | 4 | 128 (88) | 46 (74) |
| TPA* | 2 | 2 | 17 (4) | 16 (0) |
| TPO | 2 | 2 | 687 (69) | 32 (0) |
| TRAIL | 2 | 2 | 12 (8) | 5 (0) |
| VEGF* | 2 | 2 | 0 (0) | 1 (0) |
| vWF^†^ | 2 | 2 | 49 (10) | 20 (0) |

Table displays the mean value reported for biomarkers among fatal Ebola infections. All study authors were contacted regarding raw data requests. Standard deviations were calculated where multiple studies reported measured values. All measured values reported as pg/msCD40Ll unless noted by * to indicate ng/ml
